# A mountain of waste created daily: a thematic analysis of environmental sustainability experiences of postgraduate intensive care nursing students

**DOI:** 10.1186/s12912-024-02347-4

**Published:** 2024-09-27

**Authors:** Fredrika Sundberg, Heather Baid, Åsa Israelsson-Skogsberg

**Affiliations:** 1 Research and Development Centre, Skaraborg Hospital Skövde, Region Västra Götaland, Skövde, Sweden; 2 The School of Health Sciences, University of Skövde, Skövde, Sweden; 3 School of Education, Sport and Health Sciences, University of Brighton, Brighton, UK; 4 Faculty of Caring Sciences, Work Life & Social Welfare, University of Borås, Borås, Sweden

**Keywords:** Environmental sustainability, Intensive care, Critical care, Nursing, Education, Nursing education, Waste management

## Abstract

**Background:**

The healthcare sector has a negative ecological impact, and intensive care is one of the most resource-consuming areas. Nurses have a duty to contribute to climate change reduction, design climate-resilient healthcare systems, and support individuals and communities in adapting to the effects of the planetary health crisis. It is essential to incorporate environmental sustainability into nursing education so that nurses can advocate for conscientious and ethically sustainable healthcare that benefits both patients and the planet. This study aimed to explore postgraduate intensive care nursing student experiences of environmental sustainability in clinical practice at intensive care units.

**Methods:**

Data were collected using a qualitative questionnaire, and the data were analysed using inductive thematic analysis. The participants were 24 registered nurses studying postgraduate, specialist intensive care nursing courses at four universities in the south and west regions of Sweden.

**Results:**

The results describe critical care students’ environmental sustainability experiences in one overarching theme with five subthemes. Intensive care is a challenging context in terms of sustainability, where saving lives is the number one priority. There were good and bad sustainability habits among the staff, and awareness was key to improving. Clinical supplies come in unsustainable packages, and the participants wished for better alternatives and they wanted more knowledge and education on sustainable practices. The findings also emphasized the importance of a holistic perspective throughout each patient’s pathway.

**Conclusions:**

Sustainability in intensive care units is somewhat unrecognised today, although intensive care nurses want that to change. The context where saving lives is prioritized makes implementing ecologically responsible practices a challenge. However, environmental sustainability in intensive care is feasible, with education needed for nurses to take on the responsibility of making improvements. Hospital management prioritizing sustainability is also important to support clinicians in implementing sustainable practices in intensive care units.

## Background

### Sustainable healthcare

The World Health Organization (WHO) recognises that worldwide climate change is a public health crisis of paramount significance [1]. Notably, the healthcare sector’s ecological impact would rank fifth globally among nations if assessed as an entity within the framework of environmental ramifications [2]. The adverse environmental impact attributable to the healthcare sector primarily emanates from production, consumption, waste management, and energy use requisite for the procurement of commodities and materials employed therein. This encompasses pharmaceuticals, technical apparatus, and other medical-technological equipment [3]. Numerous countries, Sweden included, have now enacted legislation to advance climate policy efforts, thereby safeguarding the environment, climate, and public health [4, 5]. These legal requirements are sanctioned by constraining the adverse environmental impact of businesses and organisations while transitioning towards sustainable practices. The United Nations (UN) formulated 17 Sustainable Development Goals (SDGs), which are interconnected, indivisible, and harmoniously address the three dimensions of sustainable development: the economic, the social, and the environmental [6]. The United Nations World Commission defined the concept of sustainable development, from which these global goals emanate [6]:

*“Meeting the needs of the present without compromising the ability of future generations to meet their own needs.”* [7].

### Intensive care and resource utilization

Intensive care aims to save human lives and admits, treats, and cares for the most critically ill patients in intensive care units (ICUs). To effectively monitor and treat patients with life-threatening conditions such as organ failure, substantial resources are necessary. The greater the consumption of materials in the care of critically ill patients, the more pronounced the environmental impact stemming from intensive care becomes [8]. A more adept and innovative utilization of the existing resources is necessary compared to the current practices, as the demands of healthcare are escalating and evolving [9]. Presently, there is a limited volume of research to guide environmentally sustainable healthcare within the context of intensive care nursing [10]. The COVID-19 pandemic highlighted the significance of intensive care for society while also emphasizing the resource-intensive nature of the operation in terms of equipment utilization and the considerable need for staff possessing the appropriate expertise.

### Environmental sustainability and carbon footprint education

The International Council of Nurses (ICN) emphasized the need for incorporating the concept of environmental sustainability in nursing, as well as the integration of knowledge about the health implications of climate change into both education and continuous professional development for nurses [11]. After graduation, it can be challenging for nurses to reconcile the demanding healthcare environment with a focus on carbon footprint reduction and implementing environmentally sustainable choices in their daily practice [12]. A carbon footprint is the amount of greenhouse gases released into the atmosphere from the production, use and waste management of an item or activity; therefore, a carbon footprint represents how much the item or activity worsens climate change. Nurses are responsible for upholding and safeguarding the natural environment by reducing climate change and assisting individuals and communities in adapting to the effects of the climate crisis. Nursing leadership is imperative for taking immediate action to design climate-resilient healthcare systems [11].

Embedding environmental sustainability into nursing education is fundamental if students are to progress in their future profession to advocate for conscientious and ethically sustainable healthcare that benefits the patients without harming the planet [13, 14]. Undergraduate nursing students assessed environmental sustainability and carbon footprint as significant aspects of healthcare, underscoring the need for their implementation into the educational curriculum [15–18]. Swedish universities vary in their progress in integrating these topics into undergraduate nursing education and postgraduate specialist nursing courses, including those used to educate registered nurses for working in intensive care. Currently, there is no national overview in Sweden. Given the resource-intensive nature of intensive care, an environmental sustainability educational intervention could be highly valuable in reducing the carbon footprint of ICUs. However, an initial assessment of intensive care nursing student experiences with environmental sustainability is required to strategize intensive care nursing curriculum recommendations.

### Aim

To explore postgraduate intensive care nursing student experiences of environmental sustainability in clinical practice at intensive care units.

## Methods

### Setting and participants

A convenience sample was designed to address the study’s aim. The target population comprised postgraduate intensive care nursing students at four universities in southern and western parts of Sweden between September 2021 and January 2023. Those who voluntarily agreed to participate were included, in total, 24 students. The median amount of time participants worked as an RN before postgraduate intensive care nursing studies was 9.5 years [range 3–23] (Table 1), and they had a median age of 37 years [range 26–53] (Table 2).

**Table 1:** The participants’ years of working experience as registered nurses (RN)

Year working as a RN	*N* = 24 (%)
0–2 Years	0
3–5 Years	11 (46%)
6–10 Years	6 (25%)
11–15 Years	0
≥ 16 Years	7 (29%)

**Table 2:** The age of the participants

The participants age	Mean 36,92 Min - Max 26–53
25–35 Years	10 (42%)
36–45 Years	10 (42%)
46–55 Years	4 (16%)

### Data collection

The data collection comprised a qualitative questionnaire developed for this study using ten open-ended questions (Table 3). The qualitative questionnaire’s face validity [19] was checked by experts in sustainable healthcare and researchers with experience in interviewing, who examined and discussed the tool and agreed on the final version (group consensus). The qualitative questionnaire was not piloted because the open-ended, exploratory questions sought qualitative, narrative responses about each postgraduate intensive care nursing student’s sustainability experience in the ICU. Using open-ended questions in a qualitative questionnaire was suitable because exploring experiences about sustainability in intensive care was the focus [20].

**Table 3:** Qualitative questionnaire

*Open-ended questions in the qualitative questionnaire with the aim to understand ICU students’ experiences of sustainability in ICU*
1. What do you see when you enter an intensive care unit, in the unit as a whole and when you are in the patient room, in relation to the concept of sustainability?
2. If you could decide, what would you keep and what would you do differently from a sustainability perspective? What issues would you like to work on in relation to sustainability?
3. What are your thoughts, views, opinions on the following points in relation to sustainability: a. Use and management of pharmaceuticals in relation to recycling or waste management. b. Technical equipment, materials and their packaging used in the daily activity c. Waste management d. Personnel staffing e. Working environment/sustainable working life
4. Has your education to become a specialist nurse in intensive and critical care prepared you to work in a sustainable way after graduation? If yes, in what way?
5. Is there anything that you miss in the education for specialist nurses in intensive and critical care in relation to sustainability?
6. Do you have any other thoughts on sustainable healthcare in intensive care units that you would like to want to share?

Each participating university’s ICU program director was informed, who then authorized the study and posted a notice about it on its digital learning platform with written information about the study and how to access the qualitative questionnaire. The participants were invited to the study at the end of their education when they returned to university from clinical practice at an ICU. The completed questionnaires were returned by email. The data included 75 pages of written narratives from the participants, containing rich and meaningful data.

### Data analysis

Inductive thematic analysis according to Braun and Clarke [21] was used, a process where coding of the qualitative data identifies recurring patterns and collates these into key themes (Table 4). For the *first step*, a common document was created where all questions and answers from the 24 participants were brought together. In the first step, two researchers (F.S. and Å.I-S.) read the text carefully and separately several times to become familiar with the data. The reading was done actively to identify patterns and meaningful units regarding the phenomenon. At this stage, notes were made for what would later form the basis of the next coding process.

The *second step* initially involved the two researchers independently organizing codes from the data generated manually by using coloured pens to indicate potential patterns. The codes were then discussed together until consensus on key elements of the text and associated codes was reached. The authors did the *third step* together; all codes were written on a blackboard, and an analysis was made of codes that together formed subthemes and themes. *Step four and step five*, an overview and review of codes and formulated subthemes and theme, ensured the thematic map was consistent with the dataset as a whole and checked the relationship of individual themes to each other and separately to summarize how the synthesized themes told the overall story about the data. Finally, *step six* was done by writing the story within and across themes.

## Results

The findings describe postgraduate intensive care nursing students’ experiences regarding environmental sustainability in one theme and five subthemes.

**Table 4:** Overview of the findings. Theme and subthemes

Theme	Subtheme
ICU - a challenging context for environmental sustainability	Acknowledging the need for awareness of good and bad habits regarding sustainability
	Unsustainable packaging of ICU supplies
	Knowledge and education on sustainable practice
	The importance of a holistic perspective throughout each patient’s pathway
	Prerequisite for a sustainable work life

### ICU - a challenging context for environmental sustainability

The participants narrated their views of the ICU as a caring place where the foundation is to save lives, which created a prerequisite for continuing life when existence is at stake. Saving lives is the number one importance, and a sustainable approach to clinical practice seems to be further down the list of priorities. The ICU setting involves a multitude of technology, equipment, consumable supplies and pharmaceutical products, which consumes a considerable amount of energy and creates substantial waste with environmental and financial implications. “*The entire intensive care system is incredibly resource-intensive in terms of materials*,* time*,* finances and social sustainability” (Participant 3).* This was seen as a context where a mountain of garbage was created every day. On the other hand, ICUs have large staff numbers compared to other hospital departments, which can be an advantage if they collectively work together to prioritize collaborating on improving sustainability topics.

### Acknowledging the need for awareness of good and bad habits regarding sustainability

Being in the ICU environment made the participants aware of the good and sometimes bad habits that the staff had in the ICUs. The good habits were acknowledged as some of the ICU nurses, in some situations, were aware of their actions and deliberately tried to minimize waste and the use of materials. The good and bad habits related to how some staff had awareness regarding sustainable issues and others lacked awareness of their own actions and of the structure of the units. However, the awareness and the good habits were outnumbered by the bad habits and unawareness.

The participants wished for more deliberated actions by the staff. They identified improvements to be made, such as routines where staff could be more mindful of equipment and disposables that each patient needed and then only collect those supplies more likely to be used to prevent throwing away unused items. The participants explicitly stated that it should be mandatory only to get the required supplies for each patient care episode. Currently, staff get supplies that *might* be needed, and therefore, often thrown away unused. Participants recommended that nurses think one step ahead and plan what specifically should be taken from the storage and taken into the patient room. They also expressed an ignorance of sustainable behaviour among the staff and underlined the importance of thinking before you act and using resources wisely.

Bad habits and unawareness were also noticed regarding pharmaceuticals. It was not unusual that the ICU nurses often prepared too much of the drugs or that pharmaceuticals for acute situations were drawn into syringes instead of being more standby and had them in the original packages. Therefore, the drugs could not be returned to the medicine room and had to be thrown in the waste bin.

*“The administration of pharmaceuticals is not well-functioning. I try to do it right*,* but it depends on time. I try not to mix too much*,* instead to mix several times*,* a bit more often instead” (Participant 1).*

They also noticed that another reason for increased use of materials was acute situations when the staff focused on saving lives. Sustainability issues were not considered during life-threatening situations when there was no time or space to reflect on sustainable choices, which initiated unreflective actions.

To promote awareness and good habits, it was important that every staff member working in the ICU took responsibility for their actions to minimize unnecessary use of products, especially toxic pharmaceuticals, and, in the long run, also waste. The participants wished that behaviour such as disposing of pharmaceuticals in regular waste could be eliminated: “*Pharmaceuticals are thrown anywhere*,* in the nearest trash bin” (Participant 19)*.

It was also noticed that waste management had a spatial connection. In the patient rooms, there were often only ordinary trash bins. Outside the patient rooms in the designated areas for waste, there were complete recycling stations with designated bins for plastic, paper, and batteries. However, walking the distance from the patient area was not doable. Therefore, recyclable waste was thrown away in the trash bin.

The participants also stated that healthcare personnel should work more digitally, with better systems to improve the structure of the units and increase environmental sustainability in everyday ICU practice. They also noticed that equipment that consumed electricity could be left on without being used, raising concerns about the need for more critical sustainability thinking.

Participants appreciated that responsibility towards proactive sustainability work had several layers, and everyone had to take a step in the right direction to make improvements. They thought it was ultimately the managers’ and the organizations’ responsibility who had the overarching responsibility. The units had thorough clinical guidelines, and following these guidelines in patient care created considerable waste. They followed the guidelines as they should; therefore, vast amounts of waste are routinely created while caring for every patient. The participants identified the need to improve the units’ structure when addressing sustainability, which they viewed as outdated.

When awareness was lacking, staff threw away unused materials while tidying up the patient room. These items could be saved and used when the patient needed the products later. Staff also put on aprons and gloves when not needed, which is merely a reflex for the healthcare staff to do so. Another example of this behaviour was the staff drinking coffee from disposable cups in the break room, despite porcelain mugs being available and other more sustainable choices, such as plates and cutlery. The participants viewed this unsustainable behaviour as coming from being in a hurry and staff just passing by the break rooms and getting a cup of coffee on the go.

### Unsustainable packaging of ICU supplies

The participants indicated that ICUs were flooded with materials and equipment, of which many products were disposables. Most of the materials were accredited as being made of plastic, and they also identified much of the packaging as unnecessarily wasteful. Almost every product that came into an ICU was individually packaged and then packaged into a group of ten or twenty pieces before being put in a box wrapped with further plastic. The participants wished for a better procurement process where purchasing was well thought out and existing contracts were reviewed and updated to be more environmentally sustainable. They wanted the packages, both the materials and size, to be discussed with nurses and clinical teams. This was a shared responsibility among the manufacturers, hospital procurers, and the ICU staff. A wish was expressed for a more transparent procurement dialogue between each stakeholder to occur regularly.

Another problem the participants stated about substandard products was that they led to breaking, tearing, or falling apart, and therefore, there was an increase in the use of these clinical supplies due to them not holding what they were supposed to do. Instead of one pair of gloves and apron, the staff used two or three pairs of gloves and two aprons.

Innovative solutions were also wanted. Recycling was recognized as one way to improve sustainability, but it was acknowledged as not being the answer to everything. Instead of recycling, they wanted modern and groundbreaking ways of increasing the sustainable practice in the units. Better materials were one aspect they suggested as improving sustainability, as they knew that decreasing the use of products and supplies is better than recycling. They also suggested reusing more products than what was done at that moment.

*“Away with all the unnecessary printers and copying papers that negatively impact the environment with deforestation and forest degradation. It is time to use modern electronics such as Tablets with automatic transfer into patients’ medical records. To reduce the use of paper would also reduce our workload. As of today*,* when we print an electronic referral and then use the fax machine to send to another unit in the hospital.” (Participant 21).*

They suggested some products they had identified as possible to reuse. The participants identified that there were several improvements that needed to be made in the ICU to improve and increase the sustainability practice.

### Knowledge and education on sustainable practice

The participants experienced that environmental sustainability issues were not at all or not enough in the curriculum for postgraduate specialist nursing education. Although challenges with finding space in the curriculum were recognized, they wanted and needed to learn about sustainability in an educational program with limited teaching hours:

*“We haven’t discussed sustainability much in the education*,* the focus is more on how we should prioritize our tasks in a technical way” (Participant 16)*.

Some participants expressed a need for more knowledge about ecologically responsible healthcare practices, and they called for an increased theoretical understanding of why sustainability matters:

*“When I undertook my nursing degree*,* nobody mentioned sustainability. This has gained ground lately*,* and I have now got an eye-opener during my course in critical care for sustainability” (Participant 20).*

The participants identified a knowledge gap for themselves and newly hired staff in the ICU. It was also clear that it was difficult being newly graduated as a critical care nurse and having high standards regarding sustainability since it was not on the top of the agenda for the clinical practice. They clearly expressed the shared responsibility among the university, the clinical unit, and themselves. Environmental sustainability was also seen as a way of being; if one practised it in private life, one also intended to be more sustainable in professional life. The habit of recycling was intertwined with the person as a personal characteristic regarding sustainability should be more appreciated and acknowledged than what it was. The discourse was clear that knowledge and critical thinking were required for sustainable intensive care nursing.

### The importance of a holistic perspective throughout each patient’s pathway

The participants emphasized that sustainability in the ICU was a multidimensional issue, including diverse aspects from modern, reflective waste sorting to caring activities with a patient perspective that included good, committed, and holistic care with the patient and relatives. In the ICU context, there were opportunities to assist patients and their families in reaching sustainable life and living conditions post-ICU.

*“If you have time to complete your tasks and take care of the patient in the best possible way*,* you will feel more satisfied that you have done a good job. This requires that you have good staffing and*,* above all*,* that you have good skills in general with the opportunity for skills development at work”. (Participant 20)*

Intensive care was described as a costly, resource-intensive service where care should be provided equitably, with the appropriate patients receiving the right level of care. It was important to ensure that there were reasonable chances that patients could leave the ICU within the shortest possible care time. Equitable care was also about catching those who needed support after discharge, where post-ICU clinics were an example mentioned as important for a holistic perspective. Knowing that patients were offered follow-up was important for finding meaning and motivation in work. To provide equitable care that was perceived as sustainable, the importance of having a critical approach throughout the different parts of the care chain was emphasized.

*“Through the education*,* I have gained a picture of what a good work environment can look like and how I want to care to feel good as a nurse and a human being” (Participant 10)*.

### Prerequisite for a sustainable work life

Due to all the challenges in the ICU context, participants perceived that the conditions for a sustainable way of working existed in the ICU, where a high staff density allowed for the prioritization of sustainability issues. The ICU had better conditions regarding sustainability issues compared to many general wards, where the staff-to-patient ratio was significantly lower. The participants felt that effective workforce management was a prerequisite for long-term sustainability, including environmental, financial, and social dimensions, to ensure ‘sustainable thinking’ (cognitive attention to sustainability) is maintained.

*“I believe in a dense staffing to ensure that the sustainability of healthcare will last in the long run and not be thinned out because it will mean that sustainable thinking will be the first thing that flies out the window “ (Participant 22)*.

The participants felt that staff working in the ICU were passionate about their work, critical, and not afraid to raise questions about treatment strategies, which was perceived as a good starting point for increased sustainability thinking. Several participants had experience working during the COVID-19 pandemic when they witnessed many nurses in the hospital resigning. “*The work environment during the pandemic has been demanding and many in the work group have needed sick leave due to high workload*,* this is of course not sustainable” (Participant 18)*.

Questions were raised about prerequisites for having a sustainable working life in ICU. A sustainable working life in the ICU was exemplified by feeling safe and trustful in collegial support, having reasonable working hours with a reasonable workload, having the possibility to leave work on time, and not having to think about work issues in my spare time. Having time allocated for ongoing discussions and reflections among colleagues was important. Continuing development opportunities and support for new employees safeguarded job satisfaction and united the staff group. Working in safe interprofessional teams created feelings of coping with more challenges. Feeling secure in their professional role awoke a sense of choosing sustainable employment/work.

*“I want them to invest in the staff*,* to make them feel safe with training and to create a safe team. This can help them cope with more things that happen at work” (Participant 4)*.

Another aspect of concern about sustainability from the participant’s point of view was worries about the future. Concerns over the low application rate for the specialist postgraduate nursing course in intensive care and other vacancies in the ICU create an unsecured future with an even greater shortage of ICU nurses and worries about who will choose to work in the ICU in the future. “*There are too few staff in relation to the number of patients we have. There are no ICU nurses applying for the advertised positions” (Participant 8)*.

## Discussion

The findings in this study revealed that environmental sustainability in intensive care settings is a complex phenomenon. Intensive care is a resource-consuming practice where critical illness and saving lives must be prioritised. However, striving for intensive care practice that is less damaging to the planet must be addressed. There is no contradiction between quality of care and environmental sustainability; quite the opposite, they are intertwined and extensively linked [22]. Quality and environmental sustainability both aim to improve public health [23]. Climate change profoundly threatens human health and well-being, and the nursing profession must decrease vulnerability to harmful consequences [11]. This requires prioritising environmental sustainability by all stakeholders, including researchers, educators, politicians, managers, nurses, and other healthcare staff. Individuals have a responsibility to practice healthcare in an environmentally sustainable manner, and employers, at an organizational level, need to be accountable for endorsing and providing resources for sustainability throughout estates and facilities, procurement, waste management and clinical practice systems.

The findings revealed that the participating students identified different hierarchical layers in waste management, which are supported in the literature [25, 26], expressed as 5Rs (Refuse, Reduce, Reuse, Repurpose and Recycle). In this study, the nurses expressed that reducing waste is more important than recycling, although recycling was considered important when the product had turned into waste. However, there were some barriers to sustainable waste management, with space being an issue. The ICUs had recycling stations but not near where the patients’ care took place. Instead, they were located far away; therefore, the waste was seldom disposed of in the incorrect bin. More innovative solutions should be implemented in the ICUs to facilitate sustainable waste management. It was also acknowledged that the hospital had outdated regimes that did not simplify sustainability. It is a common misperception that sustainable interventions are costly due to a lack of knowledge regarding this area. Instead, it is quite the opposite: saving resources and reducing wasteful consumption reduces financial costs and environmental impact [24], which was evident for the participating nurses.

Unfortunately, environmental sustainability is considered a bonus, an add-on activity rather than a priority, since staff shortages, budget deficits, and supply chain interruptions overshadow sustainability [24]. When acute situations appear in the ICU, sustainability guidelines must be present—otherwise, nothing will change. The participants in this study felt they were responsible for environmental sustainability and wished that their present and future colleagues also share this vision. They expressed a need for more education on sustainability. To make that feasible, it is crucial to create awareness of this subject early in the nursing profession, and integrating sustainability in all levels of nursing education is necessary. As the findings revealed, this differs today; some universities have embedded sustainability in postgraduate intensive care nursing education, and some have not. Sustainability needs to be contextualized and can be integrated into every lecture and seminar if educators adopt a sustainability lens while teaching any ICU nursing topic. For example, educating nurses about an ICU investigation or intervention can consider opportunities to address the 5 R’s (refuse, reduce, reuse, repurpose and recycle) while teaching about this clinical activity. Environmental sustainability must also be on the agenda when curricula are revised to align with the ICN’s statement of incorporating it in nursing [11]. If we do not start early in nursing, the necessary widespread change to stop damaging the planet through healthcare practice may never happen.

### Strengths, limitations and final conclusions

The consolidated criteria for reporting qualitative research (COREQ checklist) [27] was used to ensure a rigorous approach while designing and implementing the study’s data collection and data analysis. Lincoln and Guba’s [28] trustworthiness model informed the delivery and reporting of this qualitative questionnaire project to ensure credibility, transferability, dependability and confirmability of the findings and conclusions. To enhance credibility, the thematic analysis was performed individually before discussing the findings together until consensus was obtained. This to enhance the credibility. When the themes emerged, the third researcher read the whole analytical interpretation, followed by all researchers discussing the findings as a whole until there were no further questions aiming to faciliate a critical stance to ensure rigor [29] and to minimize preconceptions. A limitation of the study is that it reflects the views of postgraduate intensive care nursing students in Sweden, which may not be transferrable to other nations with different procurement, waste management and sustainability opportunities. However, strengths included that the questionnaire responses provided a range of perspectives across four universities with insightful reflections that led to themes and recommendations that helped to fill a gap in the literature about environmentally sustainable intensive care nursing practice.

## Data Availability

No datasets were generated or analysed during the current study.
